# Approaches to cervical spine mobilization for neck pain: a pilot randomized controlled trial

**DOI:** 10.1186/s12998-020-00348-z

**Published:** 2020-11-18

**Authors:** Claire Lagoutaris, Justin Sullivan, Michelle Hancock, Andrew M. Leaver

**Affiliations:** The University of Sydney, Faculty of Medicine and Health, Sydney, NSW 2006 Australia

**Keywords:** Mobilization, Neck pain, Spinal manipulation, Manual therapy

## Abstract

**Study design:**

Pilot randomized controlled trial.

**Background:**

Better understanding of the relative effectiveness of different approaches to cervical spine mobilization has been identified as a research priority in manual therapy practice. Two distinct approaches to the practice of mobilization have emerged in recent years, based on different reasoning models for selection of mobilization techniques. The objective of this pilot study was to assess feasibility aspects for a future randomized clinical trial by exploring short-term pain and disability outcomes after a single treatment with pragmatic versus prescriptive approaches to cervical mobilization for people with recent-onset neck pain at 48-h follow-up after randomization.

**Methods:**

Twenty adults with a new episode of mechanical neck pain were randomly allocated to either pragmatic or prescriptive mobilization intervention groups. The pragmatic group received a single treatment of cervical mobilization with the technique, target segment, and grade selected by their treating therapist. The prescriptive group received a single treatment of standardized mobilization with techniques similar to a previous mobilization clinical trial. Feasibility outcomes were recruitment rates, randomization audit and completion of treatment and follow-up per protocol. The primary clinical outcome of interest was disability level measured at 48-h follow-up after randomization.

**Results:**

Recruitment rates were approximately 2.5 participants per week and 100% of eligible participants were deemed suitable for treatment with cervical mobilization. There was sufficient variety in the range of pragmatic treatments selected and the data collection process imposed minimal burden on participants.

**Conclusions:**

Our results provide supporting evidence for the feasibility of a future larger scale randomized clinical trial.

**Trial registration:**

Trial registration: Australian New Zealand Clinical Trials Registry (ACTRN12616000446460). Registered 6th April 2016. https://www.anzctr.org.au/Trial/Registration/TrialReview.aspx?id=370448&isReview=true

**Supplementary Information:**

The online version contains supplementary material available at 10.1186/s12998-020-00348-z.

## Background

Cervical spine mobilization is widely used in the management of mechanical neck pain [11]. Mobilization is a manual therapy technique that involves application of low-velocity, passive inter-vertebral movements that are within the patient’s range of motion and their control [8]. It is distinguished from cervical manipulation in that it does not involve application of a rapid thrust or production of an audible ‘crack’ that is associated with spinal manipulation. There is some evidence that cervical spine mobilization provides small improvements in neck pain at short term follow-up [14] but limited evidence of clinically significant outcomes in the longer term [8]. Further investigation of mobilization has been highlighted as a research priority by the Cochrane Back and Neck Group, as potentially serious adverse events are associated with manipulation [8].

One of the barriers to better understanding the efficacy of cervical spine mobilization is the diversity of techniques and approaches employed. Cervical spine mobilization is practiced by a range of different disciplines including physiotherapists, chiropractors, osteopaths and some medical practitioners. There is considerable diversity between and even within these disciplines and to date there have been few head-to-head clinical trials that have directly compared different mobilization techniques or approaches [8]. This type of trial is important because it is not known whether certain approaches to manual therapy are more effective than others.

In clinical trials of mobilization to date there have been two distinctly different approaches to the way that mobilization has been applied. In many mobilization trials the approach has been pragmatic, and decisions related to the choice of technique and dosages were left to the judgment of the therapists in the trial [9, 10, 13]. This approach is consistent with traditional manual therapy practice in which techniques are individualized and determined by careful assessment of spinal pain and movement dysfunction and targeted accordingly. One disadvantage of this approach is that the treatment being tested is not standardized. It also is not possible to easily transfer the results of pragmatic trials into the clinic as there is often little detail provided about the treatments that were performed. In contrast, the other approach to mobilization seen in clinical trials is prescriptive, in which all participants receive a single standardized technique with standard dosage [3, 6, 7, 18].

The two different approaches to applying cervical spine mobilization in clinical trials are reflected in the two distinctly different models of clinical reasoning for spinal manual therapy [1]. These models include the more traditional ‘segmental clinical decision-making model’ that is based on identifying a dysfunctional spinal segment and using manual therapy targeted at this segment to improve mobility and reduce pain. This is contrasted with the more recently developed ‘responder clinical decision-making model’ that is based on classifying individuals into subgroups and identifying patients who are most likely to respond to manual therapy. Classification of responders under this model is based on outcome data from clinical studies that have informed the development of clinical prediction rules [1].

Clinical application of these two different clinical decision-making models results in distinctly different approaches to mobilization of the cervical spine for neck pain and may lead to different clinical outcomes. The responder model is well aligned with a prescriptive ‘one-size-fits-all’ approach in which selection of the right patient is deemed more important than selection of the right technique. In contrast, the segmental model is more aligned to a pragmatic approach to mobilization where the technique and dose are individualized according to assessment of pain and movement dysfunction.

There is reason to hypothesise that different approaches to cervical spine mobilization might result in different outcomes for neck pain. Most cervical mobilization trials to date have compared mobilization to other treatments rather than a placebo or usual care control. Comparison of the within-group changes across these trials demonstrates quite variable effects from pragmatic [9, 10, 13] and prescriptive [3, 6, 7, 18] cervical spine mobilization. This variability has not however been explored to date in a head-to-head randomized trial of pragmatic versus prescriptive approaches. This means that the best available evidence about the relative benefits of each approach is limited.

The aim of this study was to explore aspects of feasibility for a larger scale randomized controlled trial that will compare two different approaches to cervical spine mobilization for neck pain. The proposed larger scale study will determine whether a pragmatic approach to mobilization, where the technique and dosage are selected by the therapist, is more effective in reducing disability related to neck pain at short-term follow-up than a more prescriptive approach where the technique and dosage are predetermined.

## Methods

**Fig. 1:**
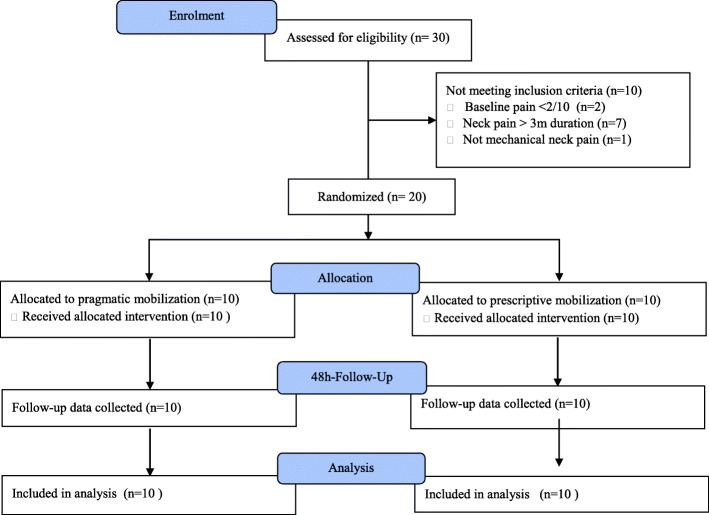
Consort flow diagram AToM Neck Pain Study

This study was a pilot parallel-groups RCT (randomized controlled trial) with participants allocated equally into two cervical mobilization intervention groups without blinding of the treating clinician (Fig. 1). Participants were recruited through advertisements that were placed on university noticeboards and social media, and interested participants were screened for exclusion via telephone using a checklist of inclusion and exclusion criteria.

Eligible participants attended the university laboratory clinic for baseline assessment, treatment and follow-up assessment. Baseline assessment included self-report questionnaires and physical examination. Following baseline assessment, participants were randomized to either a pragmatic or prescriptive intervention group and received a single treatment with cervical spine mobilization. Late randomization was used, following assessment by the treating practitioner to allow the practitioner to exclude patients they deemed unsuitable for treatment with cervical mobilisation. Outcome measurements were taken immediately after treatment and at 48-h follow-up.

The study was approved by the University of Sydney Human Research Ethics Committee (Project 2016/222) and was registered with the Australian New Zealand Clinical Trials Registry (ACTRN12616000446460). Informed consent was obtained in writing from all participants prior to participating in this trial and the rights of the participants were protected.

### Participants

Participants were aged 18 to 60, with an episode of recent-onset neck pain, defined as a primary complaint of pain of less than 3-months duration in the area between the superior nuchal line and first thoracic spinous process. Participants had mechanical neck pain that was defined as pain between 2 and 7/10 on a numerical rating scale, which was aggravated by movements or positions, and relieved fully or partly by rest. Participants were excluded if they had whiplash-associated disorder, serious pathology (such as malignancy, infection, inflammatory disorder, fracture), previous neck surgery, high risk of disability (Orebro Musculoskeletal Pain Questionnaire [16] score of > 105) or if they were incapable of completing English language questionnaires. Participants could also be excluded prior to randomisation if the treating therapists deemed them unsuitable for treatment with neck mobilization. Reasons for exclusion by the treating therapist could include specific clinical diagnosis (e.g radiculopathy, myelopathy) or clinical presentation deemed unsuitable (e.g widespread chronic pain, high level, concomitant symptoms).

### Interventions

The participants received a single treatment of cervical spine mobilization. There were no adjunctive interventions provided and participants were not provided with advice about neck care. Participants in the pragmatic group were treated with mobilization that was individualized to their clinical presentation. The treating therapist made decisions on the selection of key parameters including the treatment technique, spinal level, grade, direction, side and duration. Participants in the prescriptive group received mobilization with the same prescription that was used in a recent trial that compared prescriptive mobilization to manipulation [6] and has been used in subsequent prescriptive studies [5]. With the participant in prone, Grade IV unilateral posteroanterior pressure was applied to C1–2 on both sides and a Grade IV central posterior-anterior pressure was applied at T1–2. Each technique was applied as a 30-second bout.

Interventions in both groups were provided by 4 physiotherapists who each had postgraduate qualification in manual therapy and more than 10 years of clinical experience using manual therapy. Each therapist treated 5 participants and each therapist provided both interventions. Therapists attended a 1-h training session conducted by the principal investigator regarding the trial protocol, assessment and treatment processes.

### Randomization and blinding

Simple randomization was used to allocate participants to a treatment group. A researcher not involved with participant screening or data collection produced a random computer-generated sequence of the two treatment allocations, with an equal number of treatments for each group. In this order, they were placed in 20 sequentially numbered, sealed, opaque envelopes. The envelope for each participant was opened by the treating therapist after baseline data collection and enrolment into the study. Baseline and follow-up data collection and data analysis were conducted by researchers who were blinded to treatment allocation. Participants were informed that the trial was testing two different types of neck mobilization and were otherwise blinded to the research question. Participants were not explicitly required to be naïve to cervical mobilisation and it is possible that an experienced manual therapist might deduce the research question if enrolled in the study as a participant. Manual therapy practitioners would therefore be added as an exclusion criterion in a future study. It was not possible to blind therapists to treatment allocation. Participants and therapists were instructed to not discuss treatment allocation with the data collectors. Data collection was conducted in a separate room to the interventions. We were otherwise not able to guarantee blinding.

### Feasibility

We evaluated the feasibility of a larger well-powered RCT using criteria suggested by Leon [15] namely; recruitment, audit of randomization, and assessment procedures, as well as, completion of treatment and follow-up per protocol. We also recorded details of mobilization techniques chosen in the pragmatic group, and technique that would have been selected in the in the prescriptive group, to determine whether pragmatic and prescriptive treatments were sufficiently different to each other. Techniques were categorized as ‘passive accessory’, ‘passive physiological’ or ‘other’. The target segment grade of movement and number of repetitions and sets were also recorded.

### Clinical outcome measures

The primary outcome was the change in disability at baseline and follow-up measured with the Neck Disability Index [20] (NDI 0 to 50). Secondary outcomes included average 24-h pain and present pain index (PPI 0 to 10) measured using the Numeric Pain Rating Scale (NPRS 0 to 10), cervical range of motion (ROM^0^ composite measure of Flexion, Extension Rotation and Lateral Flexion) measured with a CROM device (Performance Attainment Associates, Lindstrom, MN, USA), and Global Perceived Effect [12] (GPE − 5 to 5). Adverse events were recorded as a negative change in NPRS or GPE at 48-h follow-up. Participants also completed a checklist of whether they experienced additional pain, headache, or other unpleasant event that they attributed to treatment, at 48-h follow-up.

### Data analysis

Participant characteristics were reported using descriptive statistics. Between group differences in treatment outcomes were analysed using independent samples t-tests. Data related to treatment decision in the pragmatic group were extracted from the clinicians’ treatment records.

The sample size for this pilot was set at 20 to allow estimates of group mean differences and variance and to test recruitment, data collection and intervention processes.

## Results

Recruitment took place in two periods between 11 April to 8 May 2016 and 10 August to 6 September 2016 due to availability of trial personnel. During this cumulative 8-weeks period, there were 30 telephone enquiries in response to the trial advertising strategy from which 20 participants were recruited (Fig. 1). The rate of recruitment was 2.5 new participants per week and recruitment ceased when the participant total reached 20. The most frequent reason for exclusion was neck pain of greater than 3 months duration. No participants were excluded by the treating therapist for being unsuitable for manual therapy, suggesting the screening questions for mechanical neck pain were aligned with the practitioners’ judgements.

All participants who enrolled in the study were randomized. All participants received treatment as per allocation. In the pragmatic mobilisation group practitioners selected passive physiological techniques in 9 (90%) cases, passive accessory in 7 (70%) and Mulligan [17] techniques in 4 (40%) of cases. Techniques were directed at the upper cervical spine (O-C2) in 5(50%) of cases, and the mid- 9(90%) and lower-cervical spine in 6(60%) of cases. Higher grade techniques (Grades III & IV) were used in 7(70%) of cases. Practitioners used 30 oscillations in 8(80%) of cases and 45 in 2(20%). Two sets were performed in 9(45%) of cases, three in 10(50%) of cases and four in 1(5%) of cases. Treatment records from the prescriptive group demonstrated that all participants were treated according to the prescriptive protocol.

Blinding of data collectors was maintained by conducting the baseline and follow-up assessments in a different room to the one in which the delivery of the interventions occurred. All participants attended the scheduled 48-h follow-up data collection session within 72 h of receiving the intervention. There were no missing data. The burden on participants was minimal with the initial session taking approximately 1 h and the follow-up session approximately 30 min.

The baseline characteristics of participants are described in Table 1. Participants in the pragmatic mobilization group were significantly older MD(95%CI) 7.8(1.7 to 13.9) years and had significantly higher baseline pain 1.2(0.2 to 2.2) /10 on a 0–10 scale than the prescriptive group. The region of neck pain was also not evenly distributed between the groups with 9 (90%) of the prescriptive group reporting lower cervical pain. Seven (70%) of the pragmatic group were taking analgesic medications compared with 1 (10%) of the prescriptive group.

**Table 1:** Baseline Characteristics

		Pragmatic Group	Prescriptive Group	*p*-value*
Age		30.7 (8.7)	22.9 (2.7)	0.02*
Gender (Female)		7 (70%)	6 (60%)	
Region of pain	Upper Cervical	1 (10%)	0	
	Mid Cervical	5 (50%)	1 (10%)	
	Cervicothoracic	4 (40%)	9 (90%)	
Concomitant pain (Headache, arm pain)		5 (50%)	5 (50%)	
Upper limb paraesthesia		1 (10%)	3 (30%)	
First episode of neck pain		3 (30%)	2 (20%)	
Using analgesic meds		7 (70%)	1 (10%)	
Workers Compensation		0	0	
Neck pain	PPI (0–10)	4.6 (1.4)	3.4 (0.7)	0.02*
	24 h average (0–10)	5.2 (1.5)	3.9 (1.5)	0.06
Disability (NDI 0–50)		11.8 (3.6)	9.3 (3.1)	0.11
OMPQ		57.4 (19.9)	63.5 (21.3)	0.5
Range of motion	Composite score^0^	301	315	0.6

Values are Mean (SD) for continuous variables and n(%) for categorical variables. PPI=Present Pain Index measured on a 0–10 Numerical Rating Scale; NDI=Neck Disability Index 0–50; OMPQ = Orebro Musculoskeletal Pain Questionnaire. *p < 0.05

The primary outcome of change in disability scores at 48 h follow-up was not significantly different (MD 3.1, 95%CI − 1.1 to 7.3) between the pragmatic and prescriptive groups. Global perceived effect of treatment was significantly higher in the pragmatic group (MD 1.9, 95%CI 0.7 to 3.1). Secondary outcomes of pain and range of motion were not significantly different between groups (Table 2).

**Table 2:** Between group differences pragmatic v prescriptive mobilisation at 48 h follow-up

		Change score at 48 h Mean (SD)	Change Score Mean difference (95%CI)
NDI 0–50	Pragmatic	6.5 (5.4)	3.1 (− 1.1 to 7.3)
	Prescriptive	3.4 (3.4)	
PPI	Pragmatic	2.2 (1.9)	0.7 (−0.9 to 2.3)
	Prescriptive	1.5 (1.5)	
P24h	Pragmatic	2.1 (1.6)	0.7 (−0.6 to 2.0)
	Prescriptive	1.4 (1.2)	
ROM	Pragmatic	29.8 (43.6)	19.7 (−14.7 to 54.1)
	Prescriptive	10.10 (27.9)	
GPE	Pragmatic	3.1 (1.4)	1.9 (0.7 to 3.1)*
	Prescriptive	1.2 (1.1)	

PPI=Present Pain Index (0–10); P24 h = Average pain score over past 24 h (0–10); ROM = Cervical spine range of motion composite score of flexion, extension, lateral flexion left and right, rotation left and right; GPE = Global perceived effect of treatment (− 5 to 5). *p < 0.05

There were no adverse effects reported at 48-h follow-up. One participant in the pragmatic group recorded a 1/10-point higher 24-h average pain score follow-up. One participant in the prescriptive group recorded a 5/50-point higher NDI score at follow-up. Three participants recorded a lower composite range of motion score (max 41^0^) at follow-up. No participants recorded a negative GPE score.

For a larger trial 96 participants would be required to demonstrate the same mean difference in disability scores seen in these results with 80% power and alpha = 0.05. Allowing for up to 20% dropout, a recruitment target of 116 would be required.

## Discussion

The results of this pilot provide supporting evidence for the feasibility of a larger scale randomized controlled trial to investigate the relative efficacy of pragmatic and prescriptive approaches to mobilization for recent onset neck pain. Analysis of 20 participants in this pilot demonstrated a significant difference in Global Perceived Effect in favour of pragmatic mobilization, however this result requires further exploration in a well-powered randomized controlled trial.

The recruitment strategy used in the pilot was simple and effective, yielding on average 2.5 new enrolled participants per week. We used an opt-in strategy whereby participants made first contact with the researchers by responding to poster and social media advertisements. This strategy separated the treating practitioner from the recruitment process ensuring that there was no inadvertent coercion to participate from the practitioner. This process could be used in a larger scale trial with local advertisements directing potential participants to a central recruitment point at which they would be referred to a local practitioner.

The recruitment advertisements used in this pilot appear to have been well targeted. Two out of every three respondents to our advertisements proceeded to randomization. The telephone screening also appeared to be very effective in identifying participants with mechanical neck pain, who might most benefit from manual therapy treatment. Our telephone screen was based on indicators for cervical manual therapy as described by Dewitte [4] et al., 2014. Practitioners were given the option of excluding potential participants prior to randomization if they felt that cervical mobilization was not an appropriate treatment for that patient. The fact that no participant was vetoed by the practitioner suggests that our screen is well aligned with the practitioners’ clinical reasoning and that this screen would be useful in a larger scale trial.

One potential problem with our trial design is the potential for overlap between each of the intervention arms. The pragmatic group had full control over their choice of mobilization technique, target segments and grade, which included the option to select the same treatment parameters that were used in the prescriptive group. Whilst passive accessory mobilization was the treatment of choice in nearly half of the decisions made, there appeared to be sufficient variability in choice of the target segment and grade in our small sample to suggest pragmatic mobilization is sufficiently different to a one-size-fits all prescription to enable testing in a controlled trial.

The interventions in this pilot were a single session of mobilization with very short-term follow-up. Whilst this is justifiable in an exploratory study with limited resources, designed to primarily assess feasibility and explore short-term clinical effects of different mobilization approaches, the results might not be generalizable to mobilization used in clinical practice. We recommend that future follow-up studies might investigate mobilization as it is more typically provided in the clinic, namely as a course of treatment. Future studies could also explore whether the differences in approaches impact on longer term outcomes. There is however, evidence that improvements in pain and disability seen following an initial manual therapy treatment though small, are cumulative over a course of treatment and have been shown to be associated with the rate and extent of recovery [19]. It could be argued that differences seen at 48 h would be expected to follow this trajectory, however, a fuller understanding of the differences between the two approaches to mobilization would be obtained by investigating a course of treatment with longer term follow-up. It is also acknowledged that mobilization is often one component of multimodal treatment. Whilst a head-to-head comparison of different approaches to mobilization is a good design to determine relative effectiveness, results might not be directly transferable to a clinical situation where mobilization is provided as a component of multimodal treatment.

The prescriptive intervention that we tested requires revision for a larger trial to ensure that it is more reflective of clinical practice and is equivalent in terms of time spent and attention provided in the pragmatic group. This is important because the non-specific or placebo effects of each arm of the trial should be comparable to enable a valid comparison between approaches. For this study we adopted the protocol from a previous study [6] where a single set of 90 oscillations were performed. The majority of pragmatic group received two or three sets of mobilizing with the opportunity for reassessment between sets. Adjusting the interventions to allow the scope for longer treatments in the prescriptive group and limiting the amount of treatment in the pragmatic group would be required to ensure that the treatments were comparable in terms of the time spent with the patient.

Outcome data are presented in this report but need to be interpreted with caution. Global Perceive Effect of treatment was rated almost two-points higher by the pragmatic group and all other outcome scores were slightly higher in the pragmatic group, though not with statistical significance. There were chance differences between the groups at baseline in some clinical and demographic characteristics that might have impacted on this result. The pragmatic group was significantly older and had a higher incidence of medication use. The prescriptive group had an over-representation of people with lower cervical pain. In a trial with a larger sample size, randomization would be expected to yield a more even distribution of these potentially confounding baseline characteristics.

There were no major adverse effects reported in either group at 48-h follow-up. The incidence of participants reporting an increase in pain (*n* = 1) or disability scores (n = 1) at 48-h follow-up was lower than rates reported in other studies [2]. One possible explanation for this is the inclusion criteria designed to selectively enrol people with mechanical neck pain and excluding those with severe (> 7/10) neck pain. We would recommend that a larger trial selectively recruit participants with mild to moderate mechanical neck pain using criteria such as those described by Dewitte [4] and closely monitor the incidence and severity of adverse effects. We hypothesise that pragmatic treatment, with the option for lower grades and adjusting the grade for patient comfort and pain provocation, might have lower rates of adverse effects than routinely prescribed high-grade accessory movements. This hypothesis could also be tested in the larger proposed study.

## Conclusions

Previous studies have demonstrated that mobilization is an effective treatment for neck pain. This pilot study demonstrates that, with adequate funding and resources, a randomized controlled trial that explores different philosophical approaches to mobilization may be feasible and advance the evidence base relevant to spinal manual therapy practice. Implications: a larger study may be conducted to investigate whether a pragmatic or prescriptive approach to mobilization is more effective in reducing pain for people with acute non-specific neck pain as pragmatic mobilization has sufficient variability in choice of target segment and grade. As this is a pilot study, the results must be interpreted with caution until this study is conducted on a larger scale.

## Supplementary Information

**Additional file 1.** CONSORT extension for Pilot and Feasibility Trials Checklist.

## Data Availability

The datasets used and/or analysed during the current study are available from the corresponding author on reasonable request.

## Abbreviations

RCT: Randomized controlled trial
NDI: Neck disability index
PPI: Present pain index
NRPS: Numeric pain rating scale
ROM: Range of motion
GPE: Global perceived effect
